# Walk Softly

**Published:** 1877-05

**Authors:** 


### WALK SOFTLY.

The tiniest pebble thrown sea-ward from the beach, causes a wavelet, whose influences are felt for unnumbered leagues out upon old ocean's bosom. The softest whisper excites vibrations in the atmosphere around us, which cease not this side the boundless ether; so the act or thought of an immortal man, however insignificant, may color a lifetime, may leave influences which shall not cease, until time shall be no longer; influences for good or ill, to millions of immortals like himself, for unending ages. These things being so, it would seem that every act should be a felt responsibility, and every thought a prayer. Let us all walk softly then, or at least with a motive and a wish for good.

A crust of bread thrown thoughtlessly by a fellow student, made Prescott, in a measure, sightless for near half a century. An ill-timed jest has severed many a warm friendship, and planted bitterness for a lifetime, where ought to have welled up the warmest, and purest, and loveliest springs of our nature. Many a time and oft, has a frown, a harsh word, an unfeeling or contemptous gesture, crushed resolves forever, which were budding to a new and changed and better life. Reader, let us all walk softly then by day and by night, at home and abroad, inasmuch as for every step in life, we must give account at the judgment.

